# Supplementary figures and images for: Physiological and molecular response mechanisms of tomato seedlings to cadmium (Cd) and lead (Pb) stress
Source: PeerJ. 2024 Nov 29;12:e18533. doi: 10.7717/peerj.18533 (PMC11610467; doi:10.7717/peerj.18533)

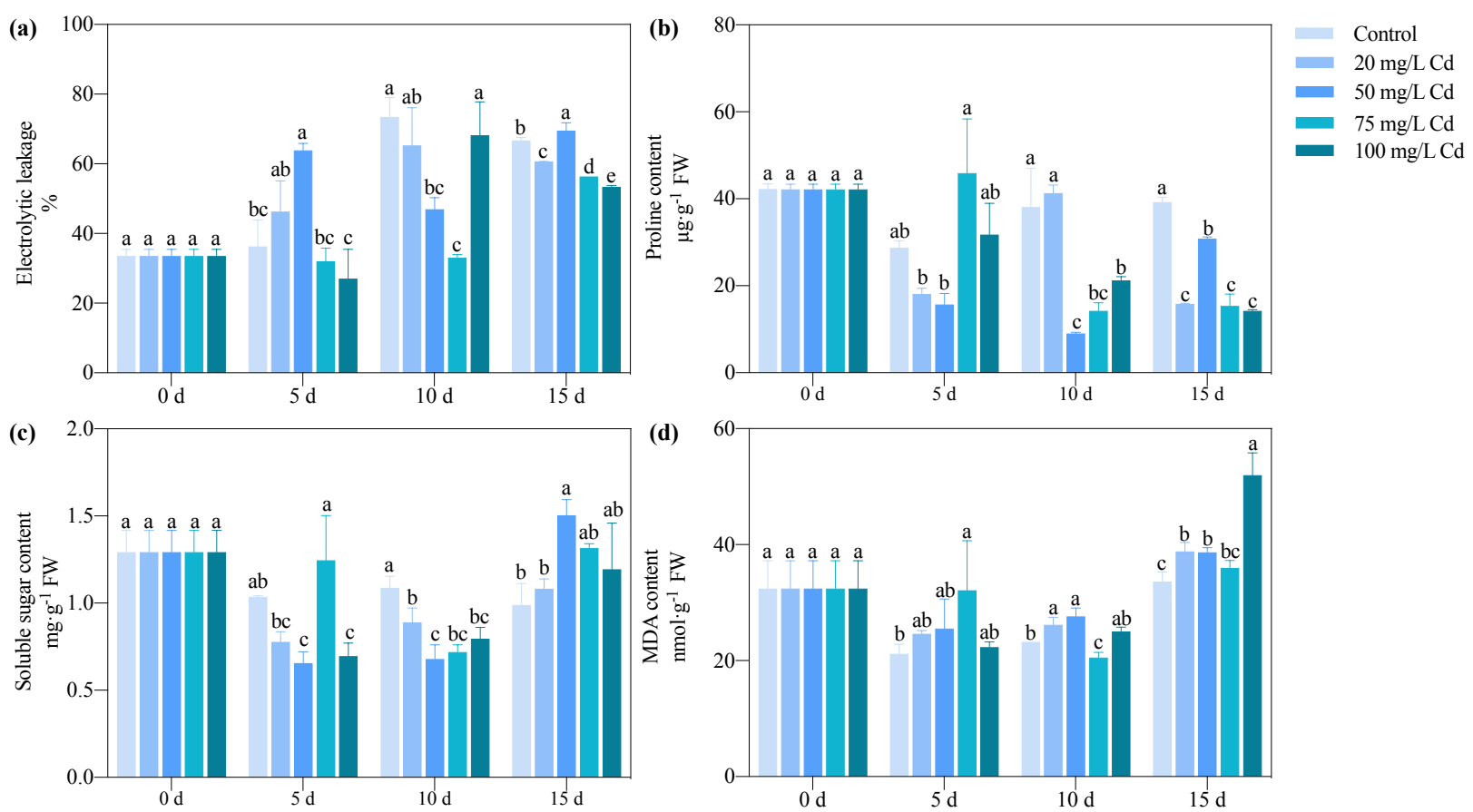

Supplement: Supplemental Information 2 — Each value is the mean ± standard error (n = 3), and the error bars represent the standa … [file peerj-12-18533-s002.pdf]

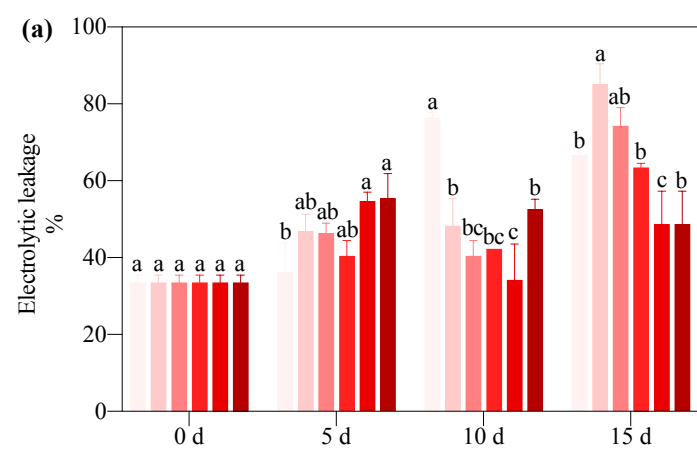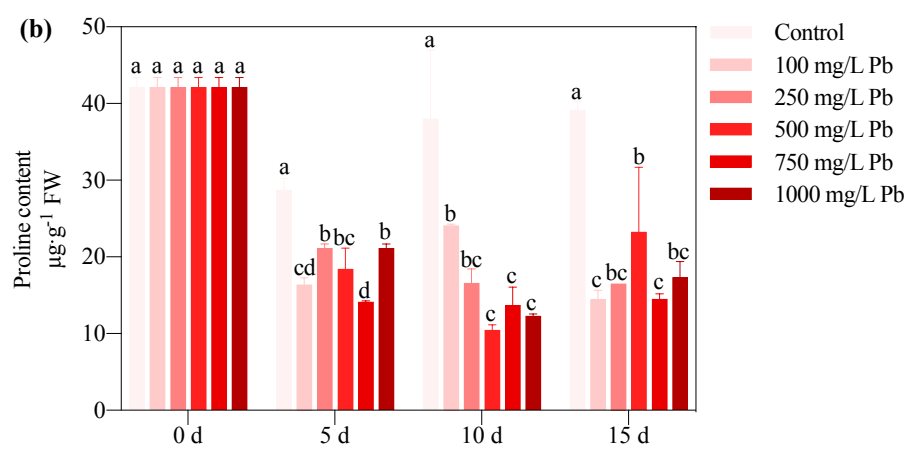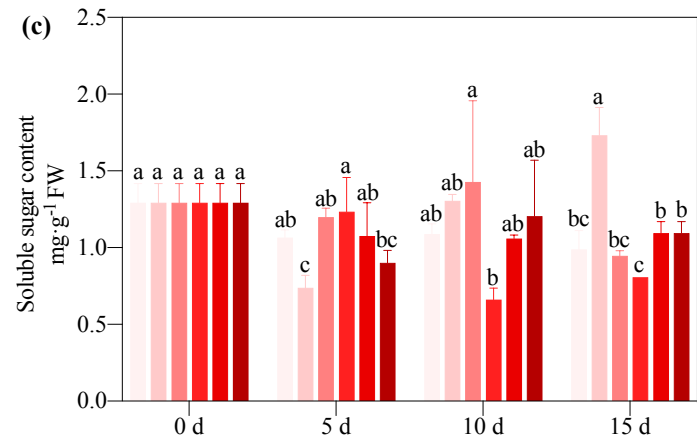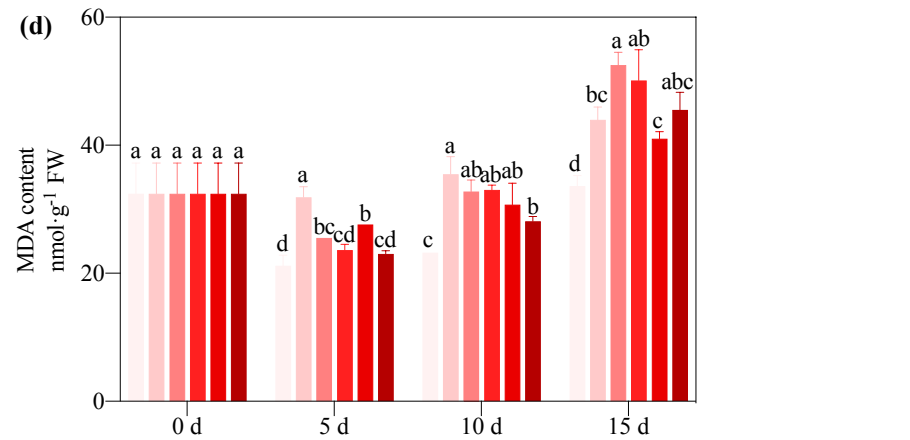

Supplement: Supplemental Information 3 — Each value is the mean ± standard error (n = 3), and the error bars represent the standard error. Bars with a different letter within a sampling date are significantly different (P < 0.05). [file peerj-12-18533-s003.pdf]

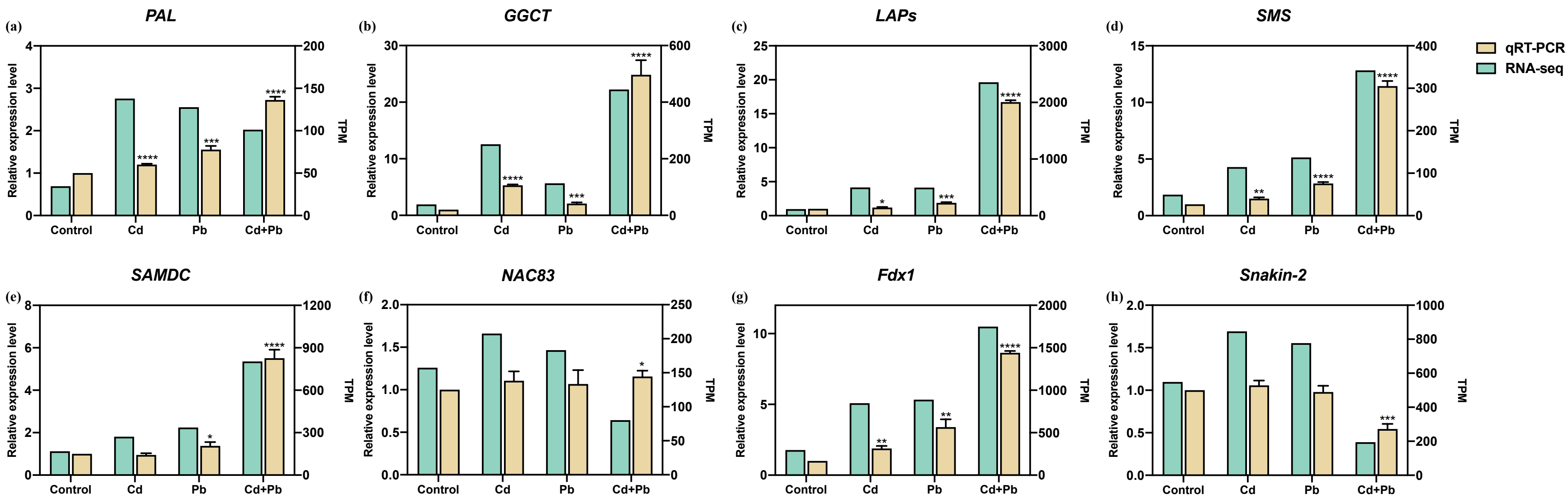

Supplement: Supplemental Information 4 [file peerj-12-18533-s004.pdf]
