# Supplementary material for: Physiological and molecular response mechanisms of tomato seedlings to cadmium (Cd) and lead (Pb) stress
Source: PeerJ. 2024 Nov 29;12:e18533. doi: 10.7717/peerj.18533 (PMC11610467; doi:10.7717/peerj.18533)
Supplement: Supplemental Information 5 — Values are means ± SD (n = 3). Values with a different letter within a sampling date are significantly different (P < 0.05). [file peerj-12-18533-s005.docx]

Table S1. Effects of different concentrations of Cd stress on growth indexes in tomato seedlings

| Treatment | Time | The increment of plant height (cm) | The increment of stem diameter (mm) | The increment of leaf length (cm) | The increment of leaf width (cm) |
| --- | --- | --- | --- | --- | --- |
| Control | 5 d | 0.95±0.07a | 0.18±0.00b | 0.33±0.12ab | 0.10±0.00b |
| 20 mg/L Cd |  | 0.60±0.00ab | 0.30±0.02ab | 0.10±0.10b | 0.20±0.14b |
| 50 mg/L Cd |  | 0.40±0.28b | 0.25±0.02b | 0.17±0.12ab | 0.06±0.05b |
| 75 mg/L Cd |  | 0.90±0.00a | 0.19±0.12b | 0.07±0.06b | 0.05±0.07b |
| 100 mg/L Cd |  | 0.85±0.07a | 0.43±0.07a | 0.30±0.14a | 0.50±0.00a |
| Control | 10 d | 1.90±0.42bc | 0.24±0.01c | 0.43±0.15ab | 0.20±0.00b |
| 20 mg/L Cd |  | 2.70±0.00a | 0.28±0.03bc | 0.25±0.07b | 0.20±0.00b |
| 50 mg/L Cd |  | 1.75±0.07c | 0.63±0.06a | 0.30±0.10b | 0.10±0.00c |
| 75 mg/L Cd |  | 1.70±0.14c | 0.60±0.02a | 0.23±0.12b | 0.20±0.00b |
| 100 mg/L Cd |  | 2.45±0.21ab | 0.36±0.06b | 0.55±0.07a | 0.55±0.07a |
| Control | 15 d | 3.20±0.35b | 0.23±0.11a | 0.35±0.07ab | 0.37±0.12bc |
| 20 mg/L Cd |  | 3.65±0.64ab | 0.13±0.05ab | 0.30±0.14b | 0.50±0.20b |
| 50 mg/L Cd |  | 2.05±0.35c | 0.04±0.02b | 0.33±0.06ab | 0.09±0.02d |
| 75 mg/L Cd |  | 1.80±0.00c | 0.18±0.00ab | 0.23±0.12b | 0.13±0.12cd |
| 100 mg/L Cd |  | 4.20±0.14a | 0.06±0.04b | 0.55±0.07a | 0.80±0.14a |

Note: The value in the table is mean±SE (n=3), and values within each row followed by the different letters indicate significant difference (*P*<0.05).
