# Supplementary material for: Physiological and molecular response mechanisms of tomato seedlings to cadmium (Cd) and lead (Pb) stress
Source: PeerJ. 2024 Nov 29;12:e18533. doi: 10.7717/peerj.18533 (PMC11610467; doi:10.7717/peerj.18533)
Supplement: Supplemental Information 6 — Values are means ± SD (n = 3). Values with a different letter within a sampling date are significantly different (P < 0.05). [file peerj-12-18533-s006.docx]

Table S2. Effects of different concentrations of Pb stress on growth indexes in tomato seedlings

| Treatment | Time | The increment of plant height (cm) | The increment of stem diameter (mm) | The increment of leaf length (cm) | The increment of leaf width (cm) |
| --- | --- | --- | --- | --- | --- |
| Control | 5 d | 0.95±0.07c | 0.18±0.00d | 0.40±0.00a | 0.10±0.00c |
| 100 mg/L Pb |  | 3.00±0.14ab | 0.43±0.05bc | 0.30±0.00ab | 0.35±0.07a |
| 250 mg/L Pb |  | 3.30±0.28a | 0.81±0.10a | 0.40±0.00a | 0.30±0.14ab |
| 500 mg/L Pb |  | 2.20±0.71ab | 0.39±0.05bc | 0.25±0.07b | 0.13±0.12bc |
| 750 mg/L Pb |  | 1.80±0.85bc | 0.28±0.11cd | 0.20±0.10bc | 0.20±0.00abc |
| 1000 mg/L Pb |  | 2.45±0.21ab | 0.55±0.08b | 0.10±0.00c | 0.13±0.06bc |
| Control | 10 d | 1.90±0.42c | 0.24±0.01c | 0.35±0.07bc | 0.20±0.00ab |
| 100 mg/L Pb |  | 3.25±0.07a | 0.56±0.03b | 0.40±0.00ab | 0.40±0.14a |
| 250 mg/L Pb |  | 3.50±0.14a | 0.58±0.04ab | 0.50±0.00a | 0.30±0.10ab |
| 500 mg/L Pb |  | 2.20±0.71c | 0.31±0.03c | 0.45±0.07ab | 0.20±0.00ab |
| 750 mg/L Pb |  | 2.25±0.35bc | 0.46±0.06b | 0.43±0.06ab | 0.15±0.07b |
| 1000 mg/L Pb |  | 3.20±0.28ab | 0.71±0.11a | 0.27±0.06c | 0.15±0.07b |
| Control | 15 d | 3.40±0.00cd | 0.23±0.11ab | 0.35±0.07bc | 0.37±0.12bc |
| 100 mg/L Pb |  | 4.70±0.14a | 0.14±0.05bc | 0.55±0.07ab | 0.90±0.14a |
| 250 mg/L Pb |  | 4.05±0.35b | 0.14±0.01bc | 0.47±0.06bc | 0.43±0.12b |
| 500 mg/L Pb |  | 3.00±0.14d | 0.37±0.08a | 0.70±0.14a | 0.30±0.10bc |
| 750 mg/L Pb |  | 3.70±0.28bc | 0.04±0.00c | 0.47±0.06bc | 0.20±0.00c |
| 1000 mg/L Pb |  | 5.10±0.14a | 0.13±0.02bc | 0.30±0.10c | 0.23±0.06c |

Note: The value in the table is mean±SE (n=3), and values within each row followed by the different letters indicate significant difference (*P*<0.05).
