# Supplementary material for: Physiological and molecular response mechanisms of tomato seedlings to cadmium (Cd) and lead (Pb) stress
Source: PeerJ. 2024 Nov 29;12:e18533. doi: 10.7717/peerj.18533 (PMC11610467; doi:10.7717/peerj.18533)
Supplement: Supplemental Information 7 [file peerj-12-18533-s007.docx]

| Gene name | Primer sequences (5’-3’) | Product size (bp) |
| --- | --- | --- |
| *Actin* | PF: CGGGAAATTGTGCGCGAC  PR: TGAGCTACTCCTGGCGGT | 87  87 |
| *NAC83* | PF: TGAGGCATTGGCTTCCGC  PR:GACGTTGAAGCTGCAACTCC | 105  105 |
| *Snakin-2* | PF: TGCAGCAAGGTGCCGATT  PR:TGCAGCAAGGTGCCGATT | 130  130 |
| *SMS* | PF: GGCCTGGCGGTGTACTTT  PR:AGGAACACTGGCCCATGC | 125  125 |
| *SAMDC* | PF: TCGTTTCCTCACCGCCAC  PR:TCCCAGCTGAGGCAGAGT | 142  142 |
| *GGCT* | PF: AGACTCTCCTCAGCCAGCT  PR:CTCTTCCAACGGGGCAGG | 100  100 |
| *PAL* | PF: TGCAGCTCCACCTACCCT  PR:GCAACGCGGCCTTCAATT | 145  145 |
| *Fdx1* | PF: TCTTGATCGGGCGGAGGA  PR: AGCAGTAACCTTTCCAGCACA | 88  88 |
| *LAPs* | PF: AGCAGTAACCTTTCCAGCACA  PR: CACCATAACCCGTGGCGT | 140  140 |
